# Impact of two nano-pesticide formulations in combating the two-spotted spider mite, *Tetranychus urticae* Koch, and their residues in cucumber fruits, *Cucumis sativus* L

**DOI:** 10.1038/s41598-025-99726-x

**Published:** 2025-05-13

**Authors:** Al-kazafy Hassan Sabry, Rania Mohamed Ahmed Helmy, Rasha Ahmed Sleem, Shaker Mohamed Abolmaaty, Aziza Hassan Mohamady

**Affiliations:** 1 Pests and Plant Protection Department, National Research Centre, Cairo, Egypt; 2 Pesticide Residue and Environmental Pollution Department, Central Agricultural Pesticides Laboratory, Agricultural Research Center, Dokki, Giza, Egypt; 3 Bioassay Research Department, Central Agricultural Pesticides Laboratory, Agricultural Research Center, Dokki, Giza, Egypt; 4 Central Laboratory for Agriculture Climate, Agricultural Research Center, Dokki, Giza, Egypt

**Keywords:** Nano-formulations, Chlorfenapyr, Emamectin benzoate, Efficacy, Residues, Cucumber, Biochemistry, Plant sciences, Zoology

## Abstract

Nano-pesticides aim to improve the efficacy and safety of conventional pesticides, but as they are still in the early stages of development, data about their environmental fate is insufficient. Therefore, the aim of this study is to compare between the conventional and nano-formulations of chlorfenapyr (CF) and emamectin benzoate (EB), by using chitosan nanoparticles as carriers and evaluating it against the two-spotted spider mite, *Tetranychus urticae* Koch. The loading capacities were 52.2 and 41.7%, respectively. The nanoparticles sizes of both chlorfenapyr (CF NPs) and emamectin benzoate (EB NPs) were 99.86 and 78.82 nm, respectively. The LC_50,_ s were 68.8, 10.8, 3.6 and 1.1 ppm for chlorfenapyr, emamectin benzoate, nanochlorfenapyr and nanoemamectin benzoate, respectively. Thus, the nano-formulations are 6- and 3-fold more toxic than the conventional ones. The reduction percentages of *T. urticae* adults reached to 98.9 and 93.8% for CF NP s and EM NP s, respectively. Dissipation kinetics have been determined and the initial deposit after one hour of application was (0.95 and 0.083) and (0.12 and 0.052) mg kg^−1^ for conventional and nano-formulations, respectively. The t _0.5_ and PHI have been determined, t _0.5_ were 0.8, 0.6, 0.9, and 0.4 days while PHI values were 7, 5, 3, and 1 day for conventional and nano-formulations, respectively. In conclusion, the nano-formulations exhibit high efficacy in controlling *T. urticae* adults and have low residue in cucumber fruits. These results cleared that the nanoformulations reduced the concentrations, the residues and increased the efficiency.

## Introduction

The cucumber, *Cucumis sativus*L. is one of the main vegetables in Egypt, which contains many important nutrients and antioxidants that positively effect human health^1^. The production of cucumber in Egypt, reached 613,031 tonnes in 2020^2^. Unfortunately, cucumbers are attacked by many pests during the agricultural season, especially the two-spotted spider mite, *Tetranychus urticae* Koch, 1836. The two-spotted spider mite, *T. urticae*infests the cucumber plant during its three growing stages: vegetative, flowering, and fruiting, and hence, it reduces its yield by 23.8%^3^. About 10% of the plants around the world infested by *T. urticae*^4^. With the heavy infestation of *T. urticae*, the adult damage can lead to defoliation, stunted plant growth, and complete plant deterioration, and even the death of plants, severely impacting both the quality and yield of affected crops. Indiscriminate and routine use of conventional pesticides on cucumber plants to suppress *T. urticae*and to enhance crop productivity may cause various ecological problems, developing resistance in mites rapidly, and failing to keep the number of mites below economic threshold levels, affecting non-target organisms and causing environmental contamination^5^. Where, more than 90% of the applied pesticides are either lost to the soil, air, or run-off and reside in agricultural products^6^. Therefore, the use of conventional formulations of pesticides on cucumber plants may have an impact on human health, especially if cucumber fruits are eaten fresh.

Nano-formulation is a new approach that can play an important role in avoiding or reducing the negative impacts of pesticide use by reducing the dose of application not only on human health and the ecosystem but also to all non-targeted organisms. It is shown to be active and more stable in different environmental conditions (sun, heat, and rain), penetrate and deliver to the target, prolong the effective duration, and reduce the runoff in the environment^7^. Therefore, the International Union of Pure and Applied Chemistry (IUPAC) ranked nanopesticides as one of the top ten emerging chemical technologies that could change the world^8^. Nano-formulation allows the use of the pesticide at lower concentrations with retention of its effectiveness against pests and ensures plant health and less environmental and nutritional contamination^9^. Chlorfenapyr is a novel class of insecticide used for crop protection. Chlorfenapyr (Challenger) is a pyrrole compound that, at the biochemical level acts as an uncouple of oxidative phosphorylation by disruption of the proton gradient. It is included in the Environmental Protection Agency (EPA) list as an alternative to organophosphorus compounds; chlorfenapyr caused 89.33% mortality of *T. urticae*^10^. Nano-chlorfenapyr was used against many pests such as *Anopheles funestus*,* Anopeles coluzzii* and *Culex quinquefasciatus*mosquitoes^11^. Chlorfenapyr nanoformulation also used against the conical snail, *Cochlicella acuta*, and the chocolate banded snail, *Massylaea vermiculata*^12^.

Emamectin benzoate has a new mode of action by affecting the GABA-gated chloride channels, stimulating the flow of chloride ions into neuronal cells with hyperpolarization, sweeping of signal transmission, and disruption of nerve impulses, which leads to death^13^. Emamectin benzoate was reported to be effective against two-spotted spider mites, less persistent in soil, and comparatively safe for beneficial organisms under field conditions^14^. Emamectin benzoate used against *T. urticae*on three crops; bean, papaya, and jute. Nano-emamectin benzoate was prepared and used a promising pest control agents against pests to overcome of pest resistsnce and improve the systemic translocation emamectin benzoate^15,16^. Nano-formulation of emamectin benzoate were used against *T. urticae*adults compred with the conventional formulation. The results found that the nanoformulation was mre effective than the normal one^17^.

This study aimed to introduce a new approach to suppress the two-spotted spider mite, decrease the spraying dosage of pesticides, and reduce toxic residues in cucumber fruits under greenhouse conditions by using two nanoformulations of chlorfenapyr and emamectin benzoate as a promising alternative strategy to conventional formulations.

## Materials and methods

### Tested pesticides

**Chlorfenapyr (CF) (Challenger 36% SC)** formulation (Huaian Glory Chemical Co. Ltd. China) was obtained from Central Agricultural Pesticides Laboratory (CAPL), Egypt. The recommended field rate of chlorfenapyr by the manufacturers was 200 cm^3^/feddan (180 ppm). The nano-formulation is prepared at one/a fifth of the conventional formulation rate.

**Emamectin benzoate (EB) (Evocut 5.7% WG**) formulation (Zhejiang Sega Science and Technology Co. Ltd. China) was obtained from CAPL). The recommended field rate of emamectin benzoate by the manufacturers was 80 g/feddan **(**15 ppm). The nano-formulation is prepared at one/a fifth of the conventional formulation rate.

## Preparation of Chlorfenapyr and Emamectin benzoate nano-formulations

Chlorfenapyr and emamectin benzoate nanoformulations were prepared^18^. High molecular weight chitosan is used as a carrier for the active ingredients of chlorfenapyr and emamectin benzoate. Chitosan (one gram) dissolved in a 2% acetic acid solution and stirred for 30 min using a magnetic stirrer to ensure chitosan completely dissolved. After that, the acidified chitosan solution was sonicated until converted into transparent. Then a 0.8% (w/v) tripolyphosphate solution containing one–fifth ml of chlorfenapyr or emamectin benzoate pesticide was added and stirred for 20–30 min. The suspension was centrifuged at 10,000 rpm for 30 min, and the pellet was collected and lyophilized. The obtained particles were photographed by a scanning electron microscope (SEM) to investigate the morphology of the prepared nanoparticles. The loading capacity (LC) of the two prepared nano-pesticides on the chitosan carrier was determined^10^. Nano formulation (30 mg) dissolved in 50 mL of acetonitrile, and shaken overnight at a constant temperature. Then, the solution was filtered, and the mass concentration of LC in acetonitrile was examined by HPLC (Agilent 1260 system with a WATO 45905 C18 column) under a detection wavelength of 278 nm. LC of the tested nanoparticles calculated by the following equation:

## Characterization of Emamectin benzoate and Chlorfenapyr nanoparticles

### **Particle size determination**

The average diameter, the size distribution, and zeta potential of samples were measured by using a particle size analyzer (Nano-ZS, Malvern Instruments Ltd., UK). For measuring zeta potential, the sample was sonicated for 30–60 min. just before assessment.

## Field emission scanning electron microscopy (FE-SEM)

**Fig. 1:**
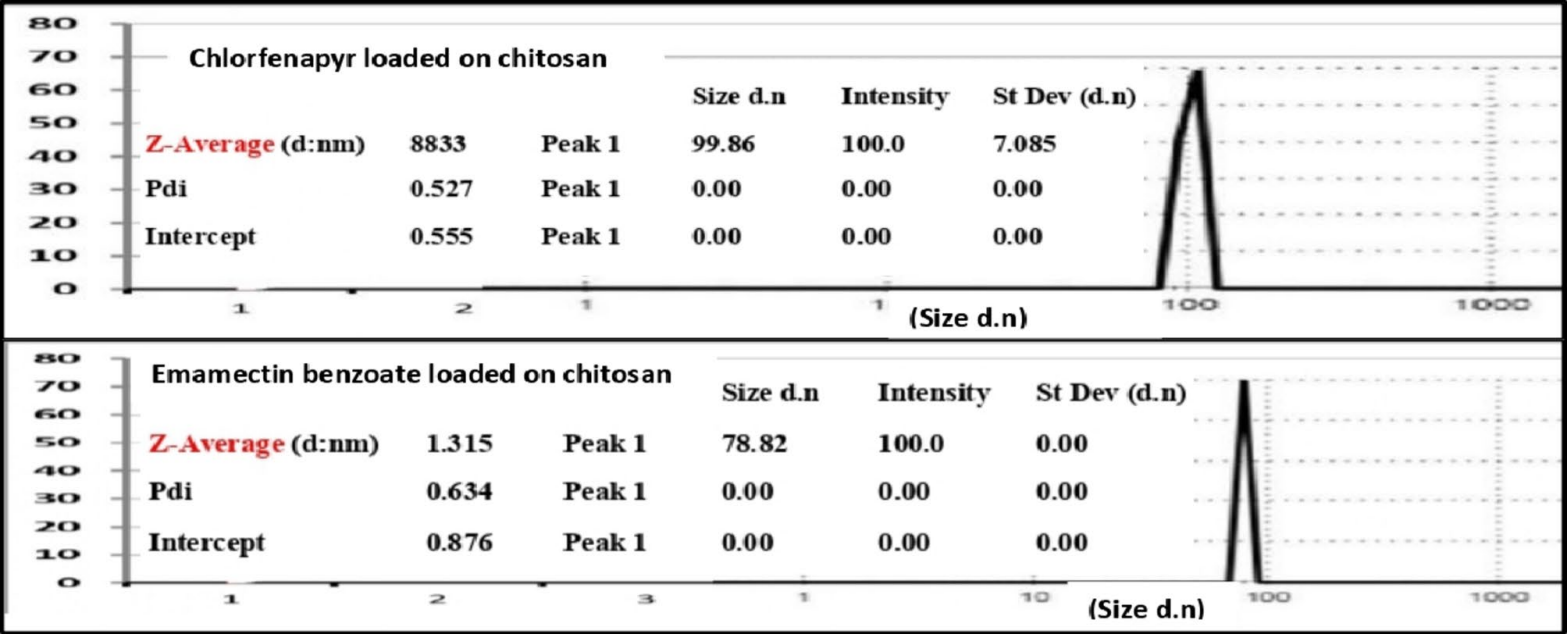
Particle size of chlorfenapyr and emamectin benzoate.

Chlorfenapyr and emamectin benzoate nanoparticles were photographed under scan electronic microscope (SEM) This work was carried out at Nanomedicine & Tissue Engineering Lab., Medical Research Center of Excellence (MRCE), Ceramic Department (Biomaterials Group), National Research Center (NRC) (Fig. 1).

**Fig. 2:**
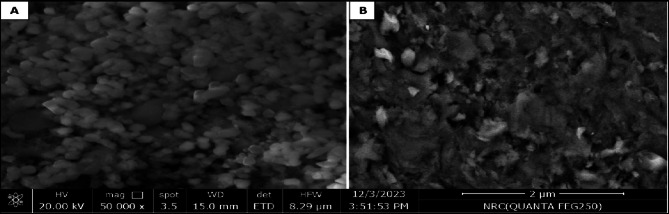
Chlorfenapyr (**A**) and emamectin benzoate (**B**) nanoparticles under SEM.

(Fig. 2)

## Tested insect

The two-spotted spider mite, *Tetranychus urticae* has left to cause randomized infestation in the tested area grown by cucumber plants. The adults of mites were used to evaluate the efficacy of the tested pesticides.

### Semi-field bioassay experiment

The seedlings of *Cucumis sativus L.*were transferred in May 2024 (Agriculture Research Center Farm, Giza, Egypt), and the irrigation and fertilization were performed under semi-field conditions according to the crop schedule. The temperature of the experimental site ranged from 26 to 39 ºC with a relative humidity of 35–75% during the experimental period. The experiment was designed as a plot area of 42 m^2^and 0.2 m^2^distance between each plant. Seven plots (6 × 7 m^2^ each) were used for each pesticide treatment. The application of pesticides was conducted according to the recommended field rate. The concentrations were prepared (in ppm) and selected according to the recommended field rate of each test pesticide. Foliar spray was carried out.

Three concentrations of each pesticide were used; chlorfenapyr (CF) is used at 180, 90, and 45 ppm, while nano chlorfenapyr is used at 36, 18, and 9 ppm. Emamectin benzoate (EB) was used at 15, 7.5, and 3.75 ppm, while nano emamectin benzoate was used at 3, 1.5, and 0.75 ppm. Three plots were used for each formulation (three replicates for each concentration) and another one as a control (sprayed with water). Plots were sprayed three times at a one-week interval with the tested formulation using a knapsack sprayer motor (20 L).

### **Efficacy of conventional and nano-formulations of both chlorfenapyr and emamectin benzoate against*Tetranychus urticae*adults populations under semi-field conditions**

To evaluate the efficacy of the tested pesticide formulations in suppression of the *T. urticae* individuals, thirty samples of cucumber leaves were randomly collected from each plot of each concentration (ten leaves for each replicate) before and after 24 h of all treatments. The leaves were placed in paper bags and transferred to the laboratory and examined using a binocular microscope. The numbers of *T. urticae*individuals were counted in each replicate. The corrected efficacy percentages were calculated according to (Henderson and Tilton)^19^ as follows:

Where:

No. = Number of *T. urticae* individuals T = Treatment Co. = Control.

The reduction percentages of mite individuals were calculated as follows:





The mortality percentages of adults were calculated after 24 h of treatment and corrected as compared to the control. Then, the corrected percentages were analyzed by the Proban Software Program (Version 4.4) to estimate the median lethal concentration (LC_50_) value for each test formulation.

## Pesticide residues and dissipation study

### Samples collection

To determine the residue levels of the tested pesticides in cucumber fruits, samples were collected randomly from the lower, middle, and upper rows of bushes after one hour, 1, 3, 5, and 7 days of application with the recommended dose. One kg of cucumbers was collected from each treatment and transported to the laboratory in a polyethylene plastic bag and stored at 4 °C. Control non-treated samples were collected for comparison.

### Samples preparation, extraction, and cleanup method

Collected samples were homogenized using a food processor. The homogenate of each sample was then placed into a 50 mL polyethylene centrifuge tube and stored at −20± 2 °C until further analysis. The residues of the tested pesticides were extracted and purified using the QuEChERS method as follows^20^: ten g well-homogenized sample was weighed into a 50 mL polypropylene centrifuge tube, and ten ml of acetonitrile added to the tube. The tube was closed and shaken for one minute using a vortex at full speed. Then, mixed salts [consisting of 4 g magnesium sulfate, 1 g sodium chloride, 1 g sodium citrate tribasic dehydrate, and 0.5 g sodium citrate dibasic sesquihydrate (Interchim, USA)] were added, shaken well, and centrifuged at 5000 rpm for five minutes. Aliquots of two ml of supernatant were filtered through a 0.2 μm PTFE filter in a standard vial for analysis.

### Chromatographic conditions

#### Chlorfenapyr

Agilent series 6890 N GC equipped with µECD detector was used for separation and determination of chlorfenapyr residues using a capillary column HP-5MS, 30 m × 0.25 mm id × 0.25 μm film thickness (Agilent J&W, HP-5, Wilmington, DE, USA). Nitrogen was used as carrier gas at a flow rate of 2 mL/min, injector and detector temperatures were 300 ^o^C and 320 ^o^C, respectively. The initial oven temperature was set at 160 ^o^C, held for 2 min then ramped to 260 ^o^C at 10 ^o^C/min and held for 10 min. Identification of sample peaks was accomplished by comparing sample and pure standards peaks using the NIST library.

### Emamectin benzoate

High-performance liquid chromatography (Agilent HPLC-1260) using a diode array-detector set at a wavelength of 260 nm was used to determine emamectin benzoate residues. The separation was performed on a column Eclipse XDB-C18 (250 mm × 4.6 mm id × 5 μm), and the mobile phase was acetonitrile/methanol (70:30, v/v) at a flow rate, of 0.8 mL/min. These conditions resulted in good separations and high sensitivity. Chemistation software was used for data analysis.

### Recovery measurement

To evaluate the accuracy of the analytical method, recovery measurements were carried out by spiking control samples of cucumber fruits with a known amount of each pesticide standard solution at three levels (0.01, 0.5, and 2.0 mg kg^−1^), and the recovery percentages (%) were calculated. Precision was studied through the repeatability, expressed as RSD (%). The limit of detection (LOD) and the limit of quantification (LOQ) were measured to determine the method sensitivity^21^.

### Kinetic studies

The degradation rate was calculated mathematically^22^.

following the first-order kinetic using common logarithms as follows:

Log *R* = log *R*0–0.434 *Kt*.

Where:

*R*0 – residue level at the initial time (zero time) *R* – Residue level at the interval (days) after application.

Kt– degradation rate constant at the successive intervals in days.

The half-life value (t _0.5_) is calculated mathematically according to (Moye et al. 1987) equation:

t _0.5_ = Ln_2_/K = 0.6932/K.

### Statistical analysis

Results were analyzed by one-way analysis of variance (ANOVA) via Randomized Complete Block Design (RCBD) (F test). The differences among means were determined at the least significant differences (LSD) using the Costat Software program^23^. Regression analysis was used in this study.

## Results

### Nano pesticides characterization

Chlorfenapyr and emamectin benzoate which were recommended to use against the two-spotted spider mite, *T. urticae* were prepared in nano-form using chitosan nanoparticles.

The nanoparticle (NPs) size of the prepared pesticide formulations was investigated by SEM. As shown in Fig. 2, SEM images showed that the particle sizes of chlorfenapyr and emamectin benzoate were in the range of 362–550 nm and have similar shapes. As well, the loading capacity (LC) of chlorfenapyr and emamectin benzoate on chitosan nanoparticles reached 52.2 and 41.7%, respectively. This suggests that both of them were successfully loaded onto the chitosan nanoparticles. In general, the pesticide-loading capacity directly affects the field application rate of pesticides.

### **Toxic impact of the tested pesticide formulations against*T. urticae***

According to the results in Table 1, the nano-formulations of the two tested pesticides decreased the median lethal concentration (LC_50_) values of *T. urticae* adults more than that of the conventional formulations under greenhouse conditions.

**Table 1:** The toxic impacts of conventional and nano-formulations of Chlorfenapyr, and Emamectin benzoate against *Tetranychus urticae*.

Formulations	Mortality Percent			Slope ± SE	LC_50_ values in ppm (CL)
	C1	C2	C3		
CF	90.5	55.3	34.2	2.7 ± 0.3	68.8 (58.9–78.5)
CF NPs	97.8	84.1	35.5	4.2 ± 0.5	10.8 (9.6–11.9)
EB	93.1	68.2	54.6	2.1 ± 0.3	3.6 (2.6–4.5)
EB NPs	89.7	48.6	32.9	2.8 ± 0.3	1.1(0.9–1.2)

C: Concentration, SE: Standard Error, CL: Confidence limit.

The LC_50_ values were 68.8, 10.8, 3.6, and 1.1 ppm for chlorfenapyr (CF), nano-chlorfenapyr (CF NPs), emamectin benzoate (EB), and nano-emamectin benzoate (EB NPs), respectively. Although the concentrations used of nano-formulations are one–fifth of the conventional formulations, the nano-formulations are more effective than the conventional formulations in both tested pesticides. Nano chlorfenapyr showed six times higher efficiency than chlorfenapyr. While nano emamectin benzoate showed three times higher efficiency than emamectin benzoate.

### **Role of both conventional and nano-formulations of the tested pesticides in controlling*T. urticae*adult populations under semi-field conditions**

Three applications of the tested formulations at the recommended field rate were carried out against the greenhouse two-spotted spider mite, *T. urticae*. The corrected efficacy (%) and percentages of adult reduction were calculated after 24 h of each application (Table 2). Results revealed that the reduction percentages of *T. urticae* individuals were 88.7, 96.2, 87.7 and 81.8% after the first application with chlorfenapyr, nano-chlorfenapyr, emamectin benzoate, and nano-emamectin benzoate, respectively. This result shows that nano-chlorfenapyr is the most effective in *T. urticae* reduction followed by chlorfenapyr, emamectin benzoate, and nano-emamectin benzoate. Regarding the results after the second application, the reduction percentages of *T. urticae* individuals were 92.6, 98.1, 73.2 and 87.7% for chlorfenapyr, nano-chlorfenapyr, emamectin benzoate and nano-emamectin benzoate, respectively.

**Table 2:** Efficacy of conventional and nano-formulations of Chlorfenapyr and Emamectin benzoate (recommended field rate) against *Tetranychus urticae* adults under semi-field conditions.

Formulations	No. of T. urticae/leaves									
	Before Treatment	After 1 st Treatment	Corrected efficacy %	% of reduction	After 2nd treatment	Corrected efficacy %	% of reduction	After 3rd Treatment	Corrected efficacy %	% of reduction
CF	245.7 ± 75.6	27.7 ± 5.3^b^	93.6	88.7	18.2 ± 5.4^b^	95.8	92.6	16.7 ± 8.7^b^	95.6	93.2
CFNPs	124.8 ± 63.7	4.8 ± 2.9^b^	97.8	96.2	2.4 ± 2.5^b^	98.9	98.1	19.1 ± 11.3^b^	90.1	84.7
EB	127.0 ± 33.3	15.6 ± 6.8^b^	93.1	87.7	34.0 ± 22.6^b^	84.9	73.2	16.8 ± 7.7^b^	91.4	86.8
EBNPs	204.6 ± 35.1	37.2 ± 7.2^b^	89.7	81.8	25.1 ± 2.8^b^	75.4	87.7	19.6 ± 9.8^b^	93.8	90.4
Control	86.7 ± 32.3	152.8 ± 37.9^a^			154.2 ± 16.3^a^			133.3 ± 20.3^a^		
*F*-values		34.4			69.1			51.9		
LSD		32.3			23.2			22.6		
df		4			4			4		
*P*-values		0.0000^***^			0.0000^***^			0.0000^***^		

*Means under each variety sharing the same letter in a column are not significantly different at *P* < 0.05.

**Fig. 3:**
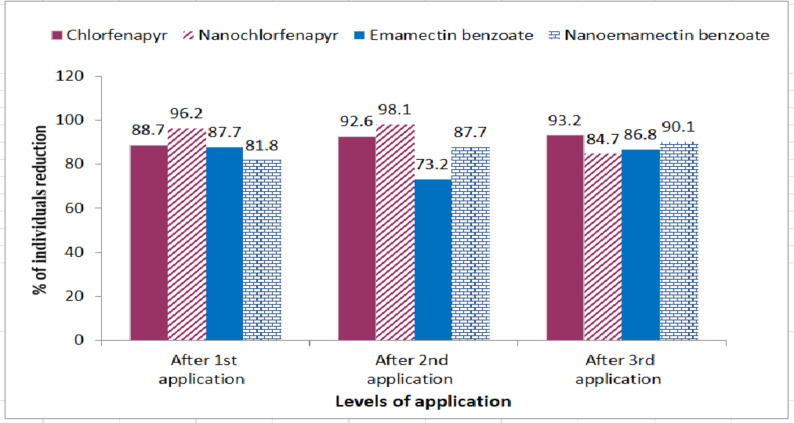
Reduction (%) of *T. urticae* adults population after application with recommended field rate of conventional and nanoformulations of chlorfenapyr and emamectin benzoate.

This result shows that the percentage of reduction after the second application was increased compared with the first application in all tested formulations except in the emamectin benzoate application. After the third application, the percentages of individual reduction were 93.2, 84.7, 86.8, and 90.4% with chlorfenapyr, nano-chlorfenapyr, emamectin benzoate, and nano-emamectin benzoate, respectively. The efficacy of both formulations also increased after the third application. Although there is a great difference in reducing the *T. urticae* population after all tested applications compared with the control (Fig. 3), no significant difference between the nano and conventional formulations used. But it is important to notice here that, the nano-pesticides concentrations are one–fifth of the conventional formulations.

## Pesticide residues and dissipation study

### Method validation

The method validation parameters linearity, limit of detection (LOD), limit of quantification (LOQ), precision, accuracy, and recovery percentage have been determined and validated according to analytical quality control and method validation procedures for pesticide residue analysis in food and feed, SANTE 11,312/2021(SANTE 2021) (Table 3). The obtained results were in harmony completely with the criteria of SANTE guideline, where the accuracy (recoveries %) was within the satisfactory range of 70–120%, and the satisfactory precision (RSD) was obtained for the two pesticides. A good linearity was observed with coefficients of determination ranging from (R^2^ **=** 0.9829 to 0.999). The recovery results ranged from 89.42 to 99.4% for chlorfenapyr and from 88.94 to 93.88% for emamectin benzoate, with RSD lower than 6% for both two tested pesticides. The LOD and LOQ values for both the two tested pesticides were 0.01 mg kg^−1^ and 0.03 mg kg^−1^, respectively.

**Table 3:** Recovery percentages and relative standard deviation (RSD %) of Chlorfenapyr and Emamectin benzoate in cucumber.

Spiked Levels (mg kg^−1^)	Chlorfenapyr		Emamectin Benzoate	
	Recovery % ± SD^*^	RSD %	Recovery % ± SD^*^	RSD %
2	99.4 ± 0.28	2.8	90.89 ± 0.08	4.1
0.5	89.42 ± 0.043	5.3	93.88 ± 0.01	4.1
0.01	92.6 ± 0.001	2.5	88.94 ± 0.004	5.4

^*^SD: Standard deviation.

Thus the obtained values of validation parameters indicated that the used method was suitable for the detection of chlorfenapyr and emamectin benzoate residues in cucumber fruits.

### Residual behavior and dissipation kinetic of tested pesticides

**Fig. 4:**
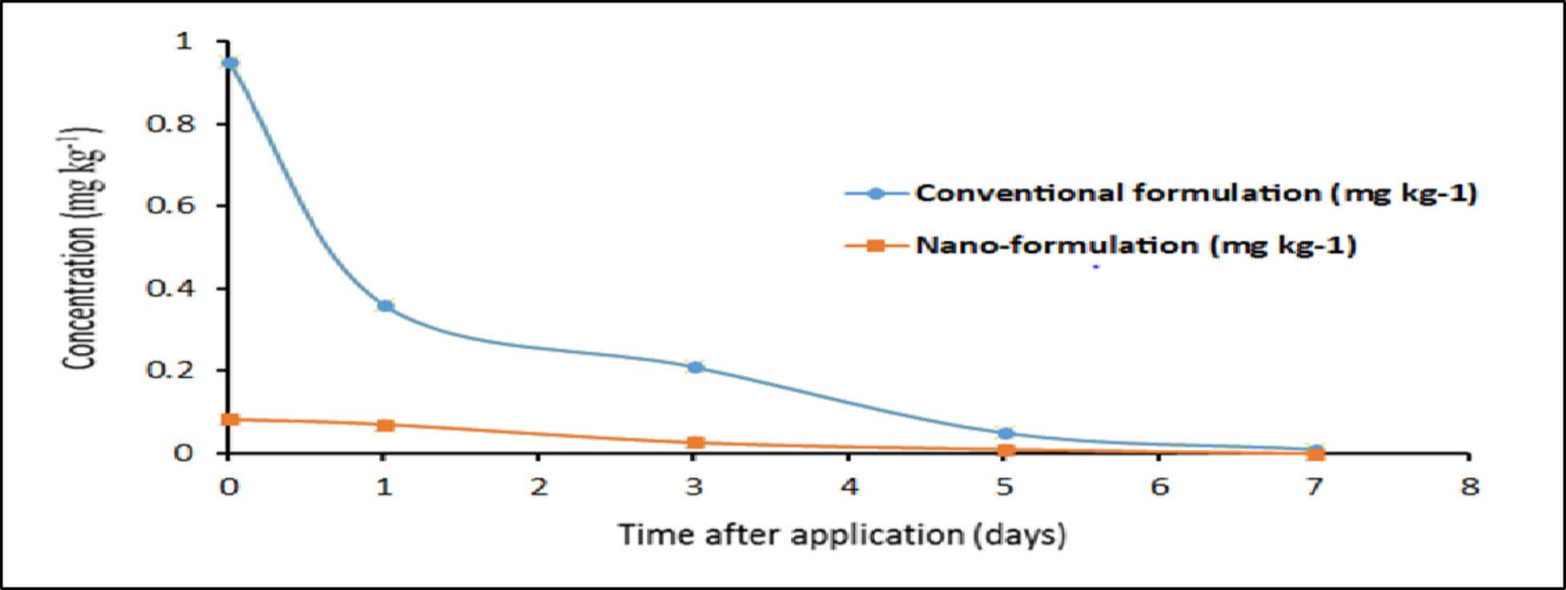
Dissipation curves of conventional and nano-chlorfenapyr residue in/on cucumber fruits.

**Fig. 5:**
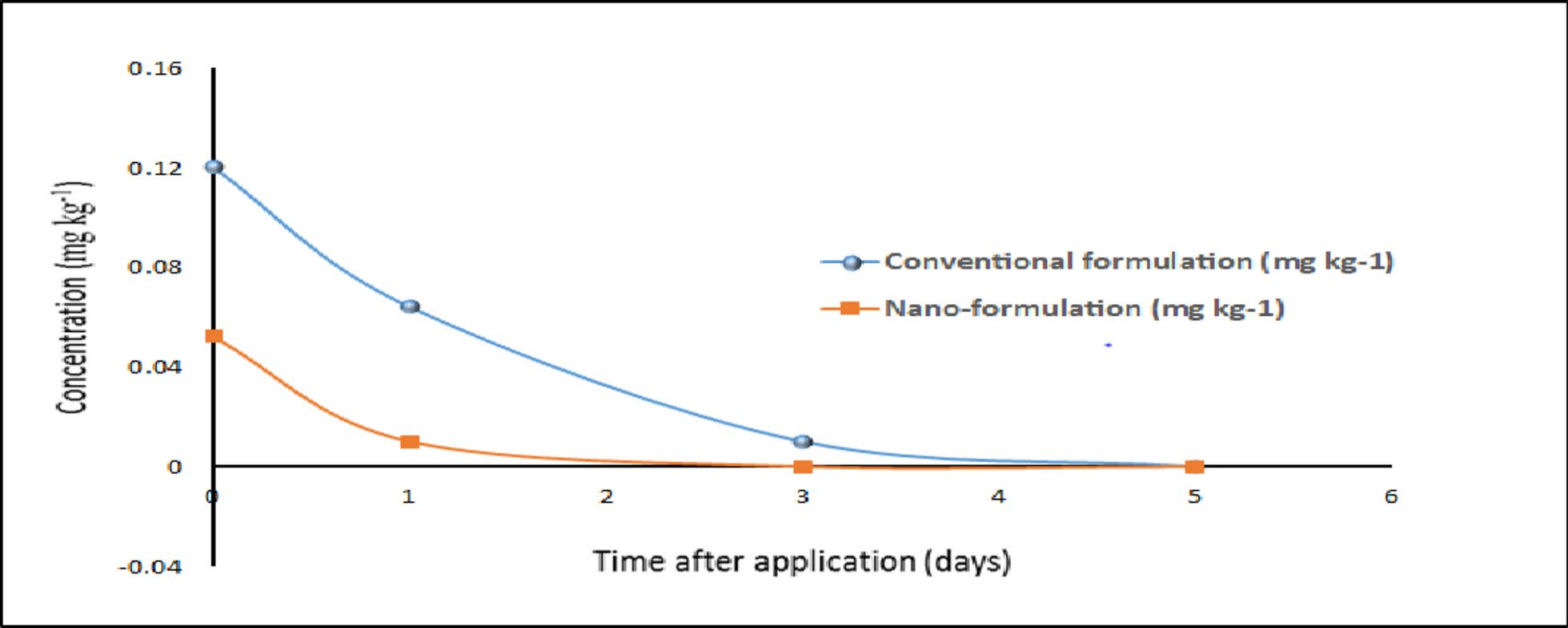
Dissipation curves of conventional and nano-emamectin benzoate residue in/on cucumber fruits.

The dissipation behavior and residue levels of the conventional and nano-formulations of chlorfenapyr and emamectin benzoate were determined in cucumber after the application with the recommended dose under greenhouse conditions. Tables (4 and 5) summarized the residue levels, rate of dissipation, Pre-harvest Interval (PHI), half-life (t _0.5_), kinetic equation, and regression coefficient (R^2^. The obtained results showed that there is a significant difference between the initial residues of conventional and nano-formulations in cucumber fruits were (0.95 and 0.083) and (0.12 and 0.052) mgkg^−1^ for chlorfenapyr and emamectin benzoate, respectively. Residues showed kinetic dissipation with time with all tested conventional and nano-formulations pesticides, were 0.95, 0.36, 0.21, 0.05, and 0.01 mgkg^−1^ at 0, 1, 3, 5, and 7 days and 0.083, 0.071, 0.027 and 0.01 mgkg^−1^ at 0, 1, 3 and 5 days for conventional and nano-formulation of chlorfenapyr, respectively. The same trend of results was observed with emamectin benzoate 0.12, 0.064, and 0.0086 mg kg^−1^ at 0, 1, and 3 days and 0.052, 0.009 mg kg^−1^ at 0 and 1 day for conventional and nano-formulation, respectively (Tables 4 and 5). The Maximum Residue Limit (MRL) for chlorfenapyr and emamectin benzoate in cucumber fruits is 0.01 mg kg^−1^, as established by the European Union^24^. Chlorfenapyr residue in cucumber fruits reached MRL after 5 and 7 days of application for nano and conventional formulations, respectively. While emamectin benzoate reached lower than the MRL after 1 and 3 days of application for nano and conventional formulations, respectively. Results showed that the residues of all tested pesticides especially nano-formulations were degraded fast with time and reached or below LOD of 0.01 mg kg^−1^ after 3 and 7 days of application for emamectin benzoate and chlorfenapyr, respectively (Figs. 4 and 5). The kinetics of chlorfenapyr residue could be described by the first-order kinetic equations y = −0.2663x − 0.0747 with R² = 0.9882 and y = −0.1907x − 1.0319 with R² = 0.9829 for conventional and nano-chlorfenapyr, respectively. The same trend with emamectin benzoate residue was y = −0.3875x − 0.8787, with *R*^2^ = 0.9912 and y = −0.73x − 1.28, with *R*^2^ = 0.999 for conventional and nano - emamectin benzoate formulations, respectively.

**Table 4:** Residual behavior of Chlorfenapyr conventional and nano-formulations in/on cucumber fruits.

Time after application	Chlorfenapyr Conventional (CF) (mg kg-1 ± SD)	RSD %	Chlorfenapyr Nano (CF NPs) (mg kg-1 ± SD)	RSD %
1 h	0.95 ± 0.03	3.2	0.083 ± 0.007	5.2
1	0.36 ± 0.04	12.4	0.071 ± 0.003	7.7
3	0.21 ± 0.07	7.9	0.027 ± 0.008	6.8
5	0.05 ± 0.004	5.9	0.01 ± 0.001	Nd
7	0.01 ± 0.002	13.1	0	Nd
Dissipation %	95		100	
MRL(mgkg-1)	0.01			
PHI (days)	7		5	
Regression Equation	y = −0.2663x − 0.0747		y = −0.1907x − 1.0319	
Regression Coefficient (R²)	R² = 0.9882		R² = 0.9829	
k	0.9		1.14	
t _0.5_ : (days)	0.8		0.6	

SD: Standard Deviation, Nd: Not detectable, PHI: Pre-Harvest Interval, R²: Regression Coefficient, k : Degradation Constant, t _0.5_: half-life.

**Table 5:** Residual behavior of Emamectin benzoate conventional and nano-formulations in/on cucumber.

Time after application(days)	Emamectin benzoate Conventional (EB) (mg kg^−1^ ± SD)	RSD %	Emamectin benzoate Nano(EB NPs) (mg kg^−1^ ± SD)	RSD %
1 h	0.12 ± 0.02	8.3	0.052 ± 0.01	3.9
1	0.06 ± 0.01	4.0	0.009 ± 0.02	11.9
3	0.009 ± 0.01	6.7	Nd	Nd
5	Nd	Nd	Nd	Nd
7	Nd	Nd	Nd	Nd
Dissipation %	100		100	
MRL(mgkg^−1^)	0.01			
PHI (days)	3		1	
Regression Equation	y = −0.3875x − 0.8787		y = −0.73x − 1.28	
Regression Coefficient (R²)	R² = 0.9912		R² = 0.999	
K	0.75		1.7	
t _0.5_ (days)	0.9		0.4	

SD: Standard Deviation, Nd: Not detectable, PHI: Pre-Harvest Interval, R²: Regression Coefficient, k : Degradation Constant, t _0.5_: Half-Life.

The half-life (t _0.5_) values of conventional and nano-formulations in cucumbers were (0.8 and 0.6 days) and (0.9 and 0.4 days) for chlorfenapyr and emamectin benzoate, respectively.

As well as the PHI values were 7, 5, 3, and 1 days for conventional chlorfenapyr, nano-chlorfenapyr, conventional emamectin benzoate, and nano- emamectin benzoate, respectively.

## Discussion

### Efficacy of nano-pesticides

The above results demonstrated that prepared insecticides in nano-form using chitosan nanoparticles could contribute to enhancing the efficacy of conventional pesticides, which may be due to their ability to improve pesticide dispersibility, penetration ability, and sticking ability with spider mite surface. Similarly, the nanoformulations of chlorfenapyr and emamectin benzoate were more effective on adult female *T. urticae*and safer than their conventional formulations^25^. Alakhdar^26^ showed that treatment by using chitosan nanoparticles produced a 94.35% mortality percentage of *T. urticae* after three days of treatment. Emamectin benzoate nanoformulation was very effective against *T. urticae*on with tomato plants^14^. In another study, In addition, Campos et al.^27^ reported that chitosan nanoparticle sustainable bio pesticide of pest control presented acaricidal activity against *T. urticae*. The nano-pesticides have many advantages compared with the conventional formulations. These advantages include improved formulation characteristics, easier application, better targeting of pest species, increased efficacy, lower application rates, and enhanced environmental safety^28^. This means that the nano-pesticides can be used with lower application rates as effective pesticides to control *T. urticae* adults under semi-field conditions and hence reduce environmental contamination. These results are in agreement with, Song et al.^29^ who demonstrated that EB@CMC@CNP performed satisfactory sustainable control efficiency to *mythimna separate* in the greenhouse. The emamectin benzoate nanoparticles were 31 times more effective than the conventional formulation of emamectin benzoate against the cotton *mealybug*,* Phenacoccus solenopsis*^30^. On the other hand, population reduction by chlorfenapyr was obtained by Reddy et al.^31^ who found that chlorfenapyr caused a 99.8% reduction in *T. urticae* population. Chlorfenapyr reduced the *T. urticae*population to 64.16 and 66.85% during the 2016 and 2017 seasons, respectively^32^. Cui et al.^33^ found that the nanoformulation of emamectin benzoate was more effective than the conventional formulation. The nanoformulation of emamectin benzoate was more stable in both low and high temptature, and high retention in plant leaves than the conventional formulation. The toxicity of nanoformulation was more than the normal formulation against *Plutella xylostella*^34^. The obtained results also showed nano-pesticdes formulations were suitable in integrated pest management^35^.

#### Residue of nano-pesticides

As expected, fast dissipation of chlorfenapyr and emamectin benzoate on cucumber could be explained due to the natural cucumber surface, the growth of the cucumber which may cause a dilution of the pesticide concentration, the chemical/biochemical decomposition, or the photolysis of these pesticides. Zhao et al.^36^reported that the dissipation of pesticides in plants is usually related to the physicochemical properties of the pesticide, the climate and experimental conditions, and the growth dilution factor. The obtained results are consistent with the findings of previous studies that worked with the same tested conventional pesticides on cucumbers or other vegetables, which recorded fast degradation of chlorfenapyr and emamectin benzoate residues in vegetables^37^. The results obtained by Abdel Ghani and Abdallah^38^ who reported that the half-life (t _0.5_) of chlorfenapyr was 0.2 days in squash. In addition, the half-life (t _0.5_) of emamectin benzoate was found to be 0.1 days in cucumber^39^, and found to be 1.3 and 1.0 days in kohlrabi and Chinese cabbage^40^. The final residues of emamectin benzoate ranged from 0.001 to 0.052 mg/kg in cabbages, with a half-life of 1.34 to 1.72 days in Beijing and Jiangsu, respectively^41^. There are few studies about the determination of nano-formulation residues in vegetables. Sharp decline of residues occurred for chlorfenapyr nano-formulation, and a comparatively slow rate of degradation was noticed for suspension concentration. The half-life values were 2.2 and 2.6 days in cabbage, while the PHI values were 3 and 5 days for chlorfenapyr nano-formulation and suspension concentration, respectively^42^. Study findings reported that the cucumber fruits could be consumed safely after 3 and 7 days of application of nano-formulation emamectin benzoate and chlorfenapyr, respectively. Using chlorfenapyr as nanoparticles improve the pesticide application and reduce the negative impact on environment^43^ On the other hand, emamectin benzoate nanoformulation increase the pest control duration against *P. xylostella*^33^.

## Conclusion

In this study, we demonstrated that both nano-formulation of chlorfenapyr and emamectin benzoate prepared using chitosan as a carrier exhibited high efficacy against *T. urticae* by six and three times more than the conventional formulation of chlorfenapyr and nano-emamectin benzoate, respectively. Moreover, through QuEChERS method, we achieved good analytical performance in terms of sensitivity (LODs, 0.01 mg kg^−1^; LOQs, 0.03 mg kg^−1^), and recovery rates ranged from 89.4 to 99.4% and 88.9–93.9% for chlorfenapyr and emamectin benzoate, respectively. Thus, this method can be used to determine very low concentrations of chlorfenapyr and emamectin benzoate residues. Nano-formulations showed fast dissipation rates; the half-lives were (0.4 and 0.6) days and (0.9 and 0.8) days for nano and conventional formulations of emamectin benzoate and chlorfenapyr, respectively. This means that the degradation rate of nano-pesticides was faster than the normal formulations. In addition, nano-formulations appeared in lower residue amounts than conventional formulations in cucumber fruits. These findings indicate that nano-pesticide formulations may be expected to contribute to improving the efficiency of conventional formulations and overcome their disadvantages. Nano-pesticides were used at low concentration. It may be clear that the use of nasno-pesticides can reduce the cost of application compared with normal formulations. So, these results recommend the use of nano-pesticides instead of the normal formulations.

## Data Availability

Raw data are avilable from the corresponding author on reasonable request.
